# Supplementary material for: Chromothripsis during telomere crisis is independent of NHEJ, and consistent with a replicative origin
Source: Genome Res. 2019 May;29(5):737–49. doi: 10.1101/gr.240705.118 (PMC6499312; doi:10.1101/gr.240705.118)
Supplement: Supplemental Material [file supp_gr.240705.118_Supplemental_file_1.zip › contigs/annotated_contigs/DB57/contig.2.DB57_length_797_mean_cov_12.5621079046.docx]

**DB57_length_797_mean_cov_12.5621079046**

```
TCCAATTTTCAGCTGCAATTGTTTTTATGTTCTTTTATGCAGGGGTTGTGAAGGCTCACTCTAAATAACACTCATCCTCAGCCAGGCTG
 >chr11:47152080-47152451 - E=2e-205
CTCTGCCAAGAGATGCTCGGAAGGACATGGATAGTTCGGGGCATGACCACACCACCCCTTCCAATCCCTGGGGCACCAAGGCTTTGTTA

TCAGGCCACAGCTGAGTAAATACTTCTCAGTGCTACTCTGTGAGGAGGGGGAAGGGAGTGAGGGACAGTGTGGGGGAAGGGAAGAAGGA

GGCAAGACATCTGACGACTTCACTCCCCATGTAGCAGTTGGTATCACATGTCACAGAAAGATTGCTACCCTACTAGGGCCATGTTCCTG

TCAAGCAAAGCCC|CA|TGAAAATGGGAGAAAATGCCTCTCCTGGCCTGGAGATCTCACAGGAGGGAAATGTGTTTCTAACAGCCATGG
              >chr11:47419912-47420340 + E=1e-242
GCCCAGCCCTCCTTCAGGCACCGGCTCTGTCCAGTAGGTACGATTATTACTCCCATTTTACATACGAGGAAACCCAGGGAAAAGAGATG

TCCCAAGGTCACACAGCCTTGCAGGGGCTTGAACCCCAATCCTGTCTAACTCCAAGTCCCATGCACTTACATATACCTGAGAGCCCCTC

CTTCCTTTTCTCCTAGGAGTTCTTCTGTACCTTCCTAGCTTGCAGGGTTGGGGCCCTGGGTGGCAAGTTGAACCATGACTGGATGGGAC

TGGAGGGCTTGGCCCTGCCTGGCTGAGGCTGCAGTCCGCAAATACCTGACTGCCCTTGCTCTTGGTTGGGCTGAGGGACTGCTGCCT
```
